# Camellia-Derived Bioactive Compounds: Research Advances and Application Prospects in Dermatology

**DOI:** 10.3390/ijms27135963

**Published:** 2026-07-02

**Authors:** Lianxin Zhang, Baoyan Dai, Hong Shen, Siyu Chen, Wenxiang Zhang

**Affiliations:** 1 State Key Laboratory of Natural Medicines, School of Life Science and Technology, China Pharmaceutical University, Nanjing 211198, China; 3225030995@stu.cpu.edu.cn (L.Z.); 2020220950@stu.cpu.edu.cn (B.D.); 1520240195@cpu.edu.cn (H.S.); 2 Yunnan Characteristic Plant Extraction Laboratory, Kunming 650106, China

**Keywords:** Camellia extract, bioactive compounds, biological activity, cosmetic application

## Abstract

*Camellia japonica* L., an East Asian species with extensive ethnobotanical use, is a rich source of bioactive metabolites including polyphenols, saponins, terpenoids, sterols, and fatty acids. These compounds have attracted significant attention in cosmetic research due to increasing demand for natural, multifunctional ingredients with antioxidant, anti-inflammatory, antimicrobial, moisturizing, and skin-brightening properties. This review summarizes the major classes of Camellia metabolites, their chemical characteristics, and mechanisms of action. Terpenoids and polyphenols, including phenolic acids, flavonoids, and tannins, exhibit potent antioxidant and anti-aging properties. Camellia saponins serve as mild natural surfactants for gentle skin cleansing, while phytosterols, amino acids, proteins, and seed fatty acids synergistically reconstruct the epidermal barrier and maintain cutaneous hydration. This review further addresses the current applications of these Camellia-derived bioactives in ameliorating photo-aging, hyperpigmentation, skin inflammation, and barrier dysfunction. Despite significant progress, key challenges persist, including incomplete understanding of biosynthetic regulation, suboptimal extraction methods, limited study of synergistic effects, and insufficient human safety data. Future studies should employ omics technologies and green extraction approaches to elucidate biosynthetic pathways, validate efficacy, and promote sustainable utilization of Camellia resources in cosmetics, pharmaceuticals, and related industries.

## 1. Introduction

**Figure 1:**
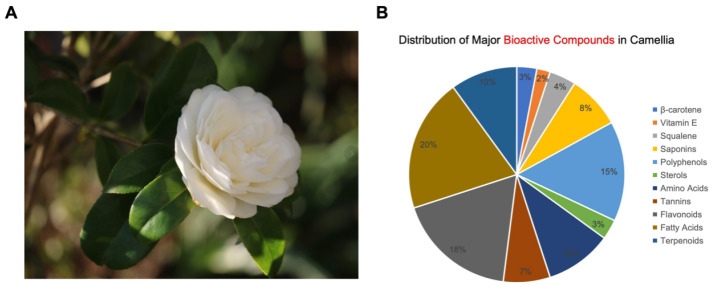
*Camellia japonica (Linnaeus)* and its major bioactive compounds. (**A**) Physical pictures of Camellia plants; (**B**) bioactive compounds of Camellia and their proportions.

*Camellia japonica* L., a representative Theaceae species native to East Asia (China, Japan, the Korean Peninsula), boasts a millennium-long cultivation and application history in China. Recognized as a traditional ornamental and multifunctional plant (Figure 1A), it provides medicinal, edible and industrial resources [1]. The entire plant (flowers, leaves, fruits, seeds, and bark) is rich in bioactive compounds characterized by unique structures and potent bioactivities. These compounds have evolved to resist environmental stresses, thereby providing a crucial material foundation for developing bioactive natural products for human applications [2]. Fueled by interdisciplinary integration of natural product chemistry, pharmacology, and dermal physiology, coupled with rising consumer demand for green, safe and effective cosmetics and health products, Camellia’s bioactive compounds have become a prominent research focus globally.

Research on Camellia bioactive compounds commenced in the mid-20th century, initially centered on seed oil extraction. Advancements in separation science and analytical characterization have enabled researchers to identify an array of compounds, predominantly polyphenols, flavonoids, saponins, terpenes, and various fatty acids (FAs) (Figure 1B) [3]. These compounds exhibit diverse bioactivities including antioxidant activity, anti-inflammatory effects, antibacterial capacity, moisturizing performance, skin brightening via pigment modulation, and anti-aging effects [4,5]. Consequently, their utilization spans cosmetic formulations, pharmaceutical compositions, and food additives.

Camellia bioactive compounds demonstrate prominent therapeutic value in dermatology and skincare. Modern research links skin aging, hyperpigmentation, inflammation and xerosis to oxidative stress, free radical damage, inflammatory cytokine dysregulation and impaired skin barrier [6]. Camellia-derived polyphenols, including catechins, epicatechin, and gallic acid, exhibit potent antioxidant activity [2]; their free radical scavenging capability, alongside inhibition of lipid peroxidation and mitigation of oxidative damage in skin tissues have been extensively documented [2], thereby delaying dermal senescence and demonstrating functional efficacy. Flavonoids, specifically kaempferol, quercetin, and isorhamnetin, possess properties relating to anti-inflammatory responsivity, epidermal lightening, and soothing effects mediated by regulation of inflammatory signaling pathways and tyrosinase inhibition, as evidenced by multiple studies [7]. Application of unsaturated FAs (particularly oleic, linoleic, and linolenic acids) from Camellia seeds promotes stratum corneum integrity and enhances moisture retention through augmented skin barrier repair [8]. Camellia saponins function not only as surfactants but also facilitate nutrient penetration into cutaneous tissues, thereby enhancing topical delivery systems [9]. Current commercial applications demonstrate that synergistic interactions among these bioactive compounds have driven the development and widespread adoption of cosmeceuticals formulated with Camellia extracts as key actives [10].

Camellia is predominantly used topically in clinical practice with rare oral administration. Flower and seed extracts alleviate acne, atopic dermatitis (AD), skin barrier damage, and promote wound healing [11]. They exert antioxidant effects and suppress inflammatory factors, thereby relieving erythema and pruritus. Orally administered extracts provide adjuvant antioxidant and lipid-regulating benefits. Toxicological assessments indicate low acute and subchronic toxicity along with the absence of mutagenicity in Camellia extracts [12]. Topical application presents minimal irritation and sensitization risks, although erythema may arise in individuals with Camellia pollen allergies [13]. Excessive oral intake may cause diarrhea in individuals with spleen–stomach cold deficiency (a traditional Chinese medicine constitution) [12], and concomitant use with anticoagulants carries a potential bleeding risk [14]. Comprehensive clinical safety data for pregnant women, lactating women, and children remain insufficient, necessitating caution. Overall, Camellia exhibits established efficacy and favorable safety for topical use, whereas high-quality clinical evidence supporting oral administration remains limited.

Currently, Camellia species and their bioactive compounds have been integrated into a diverse array of skincare products available commercially. In anti-aging formulations, high-concentration whole-plant extracts of red Camellia are commonly employed, wherein polyphenols act synergistically with Camellia ceramides to exert potent antioxidant effects and stimulate collagen synthesis, rendering these products suitable for broad skin types and effective in diminishing fine lines and ameliorating dullness and roughness [15]. In lipid-based skincare, cold-pressed Camellia seed oil serves as a core ingredient due to its lipid composition closely mimicking human sebum, emphasizing lipid-based nourishment to rapidly restore dry, compromised barriers and revitalize a lackluster complexion, particularly suited for dry and barrier-compromised skin. For sensitive skin care, formulations incorporating red Camellia essence, alongside Camellia seed oil and patented soothing actives, effectively alleviate redness, maintain cutaneous homeostasis, and repair compromised stratum corneum. These products are typically formulated without alcohol and artificial fragrances, making them appropriate for daily maintenance of compromised skin susceptible to seasonal erythema or post-exfoliation irritation [8].

Despite significant advances in Camellia bioactive research, several critical challenges persist. The content and structure diversity of bioactive compounds vary across cultivars, growth environments, and plant tissues, yet the underlying regulatory mechanisms governing these variations remain poorly characterized, hindering complicating targeted cultivation and the selection of high-quality raw materials [16]. Moreover, most mechanistic insights into bioactive compounds are derived from in vitro or animal models; their human metabolic fate, molecular targets, and long-term safety profiles still require rigorous clinical validation. Regarding extraction methodologies, conventional solvent-based methods remain predominant yet are constrained by low efficiency, residual solvent contamination, and potential degradation of thermolabile compounds. While environmentally friendly alternatives such as supercritical fluid, microwave-assisted, and enzymatic extraction demonstrate considerable promise, their optimization and industrial scalability warrant further investigation [17]. Lastly, current applications remain largely restricted to isolated components or crude extracts, whereas synergistic interactions among compounds and the rational design of multi-component formulations remain largely unexplored.

In response to these challenges, this review presents a comprehensive and systematic literature analysis of Camellia bioactive compounds reported in recent years, elucidating the chemical structures, biological activities, and mechanisms of action of the key bioactive compounds. We specifically focus on their current application status and research progress within the dermal and cosmetic sectors. Furthermore, we prospect the potential for their integrated utilization across multiple fields and delineate future research directions. This work aims to provide a theoretical framework and technical reference for the in-depth exploration of Camellia resources, the efficient utilization of its bioactive compounds, and the innovative development of related products.

## 2. Search Strategy

For this review, literature searches were performed across electronic databases including PubMed, ScienceDirect, and Web of Science. We prioritized recently published research, while also including a small number of early landmark publications to trace the historical research background. Search terms included the following: “Camellia”, “Terpenoids”, “Tea saponin”, “Phenolic Acids”, “Flavonoids”, “Tannins”, “Sterols”, “Amino Acids and Proteins” and “Fatty Acids”. Duplicate references were removed. Based on this search and criteria, the cited articles were refined for this review.

## 3. Bioactive Compounds

### 3.1. Terpenoid

Terpenoids constitute a major class of plant secondary metabolites derived from biosynthesized isoprene units, which are taxonomically categorized into four subtypes according to polymerization degree: monoterpenes, sesquiterpenes, diterpenes, and triterpenes [18]. In Camellia-derived metabolic components, terpenoid compounds comprise approximately 10% of the total compounds, distinguished by inherent volatility, lipophilicity, and unique aromatic characteristics. Modern pharmacognosy studies have demonstrated that Camellia terpenoids exhibit prominent antioxidant, anti-inflammatory, and antimicrobial bioactivities, conferring them significant application value as functional cosmetic ingredients [18]. The accumulation of terpenoids exhibits distinct tissue specificity in Camellia plants: fresh leaves are abundant in volatile terpenoids and triterpenoid structures, and tea seeds contain abundant triterpenoid components, whereas flowers, roots, and stems accumulate only trace amounts of terpenoid substances [19].

#### 3.1.1. Monoterpenes

Monoterpenes are typical volatile terpenoid components in Camellia, with limonene and myrcene serving as representative active monomers. These compounds exert direct antimicrobial efficacy against common pathogenic microorganisms associated with skin disorders, including *Cutibacterium acnes* (*C. acnes*), *Staphylococcus aureus*, and *Malassezia* species, thereby inhibiting the proliferation of skin opportunistic pathogens and reducing the risk of inflammatory skin lesions [18]. In terms of skin regulatory functions, volatile monoterpenoids can modulate cutaneous nerve conduction pathways, effectively alleviating skin erythema, nociceptive sensation, and pruritus induced by external irritation or skin damage [20]. Additionally, monoterpenoids facilitate the biosynthesis of ceramides and epidermal lipids, promoting the reconstruction of skin barrier homeostasis in compromised skin tissue and enhancing the cutaneous resistance to environmental stimuli, which is particularly suitable for the maintenance of sensitive and injured skin [21]. Furthermore, monoterpenoids participate in the regulation of melanogenesis by inhibiting tyrosinase catalytic activity, downregulating the transcriptional expression of microphthalmia-associated transcription factor (MITF) and tyrosinase-related proteins, and mediating the mitogen-activated protein kinase (MAPK)/cyclic adenosine monophosphate (cAMP) signaling cascade, thereby ameliorating UV-induced hyperpigmentation and pigmentary disorders [22].

#### 3.1.2. Sesquiterpenes

Sesquiterpenes, representing another vital class of volatile terpenoids in Camellia, share core skincare bioactivities with monoterpenes and exhibit synergistic skin-protective effects. Similar to monoterpenoids, sesquiterpenes possess broad-spectrum antimicrobial properties against skin pathogenic microorganisms, suppressing the metabolic activity of *C. acnes* and other harmful bacteria to reduce the occurrence of inflammatory acne lesions [23]. They can soothe overexcited cutaneous sensory nerves, alleviate acute inflammatory symptoms such as skin flushing and itching, and ameliorate the discomfort of damaged epidermal tissue [20]. Moreover, sesquiterpenoids assist in repairing impaired skin barriers by upregulating epidermal lipid synthesis, enhancing the structural integrity of the stratum corneum, and improving the tolerance of fragile skin to external adverse stimuli [21]. As key antioxidant bioactive compounds, sesquiterpenoids can scavenge intracellular reactive oxygen species (ROS), inhibit oxidative stress-induced skin aging, and regulate melanin metabolic pathways to improve abnormal skin pigmentation [22].

#### 3.1.3. Triterpenes

Triterpenoids represent the most abundant and functionally diverse terpenoid components in Camellia, with squalene serving as the core characteristic triterpenoid unsaponifiable lipid in Camellia oil. Squalene comprises approximately 4% of the total lipid content of Camellia oil, and its unique oxygen-carrying capacity enhances tissue oxygen utilization efficiency and blood oxygen concentration, thereby facilitating cellular metabolic renewal and contributing to tissue repair regulation [22,24]. Pharmacological studies have confirmed that squalene exhibits anti-hypoxic, anti-fatigue, and anti-aging effects, effectively delaying chronological skin aging [24]. Meanwhile, it inhibits ultraviolet-induced lipid peroxidation, blocks free radical-mediated oxidative damage to dermal tissues, and exerts significant photoprotective effects [25].

In terms of anti-inflammatory regulation, squalene inhibits the secretion of key pro-inflammatory cytokines including tumor necrosis factor-α (TNF-α) and interleukin-6 (IL-6) in the epidermal–dermal signaling axis, thereby alleviating cutaneous inflammatory responses and mitigating inflammatory skin damage [26]. Its excellent biological compatibility minimizes adverse skin reactions, effectively relieving erythema, burning sensation, and pruritus in impaired epidermal areas and improving the comfort of damaged skin [27]. As a high-efficiency emollient, squalene possesses superior skin permeability and affinity, enabling rapid penetration deep into the stratum corneum, fusion with endogenous skin lipids, formation of a breathable protective film on the skin surface, reduction in transepidermal water loss, and improvement of skin hydration and suppleness without producing oily residue [24]. Notably, squalene is non-comedogenic, neither blocking skin pores nor inducing acne lesions, enabling its universal applicability across all skin types [28].

Squalene promotes cellular oxygenation, optimizes energy metabolism and immune regulation, accelerates the renewal of senescent skin cells and the clearance of exogenous harmful substances, and comprehensively enhances skin physiological status [29]. By accelerating epidermal turnover and promoting the shedding of melanin-rich keratinocytes, squalene ameliorates skin dullness and enhances cutaneous radiance [30]. It further upregulates the synthesis of collagen and ceramides, strengthens skin barrier function, reduces water loss, and enhances skin resistance to external stressors to achieve anti-aging efficacy [31]. Additionally, squalene removes excess sebum and necrotic keratinocytes, eliminates inflammatory metabolites of *C. acnes*, and exerts definite anti-acne effects [32].

Squalene has significant therapeutic and repairing effects against multiple skin disorders. AD is characterized by congenital skin barrier defects and endogenous lipid depletion, and squalene alleviates AD symptoms through supplementing skin lipids, repairing barrier structure, providing moisturization, and inhibiting inflammatory cytokine expression [33]. It further improves xeroderma via anti-inflammatory and barrier-repairing effects, relieves skin erythema through ROS scavenging and collagen synthesis promotion, accelerates skin wound healing by modulating macrophage activity and extracellular matrix (ECM) synthesis, and mitigates UV-induced hyperpigmentation by inhibiting DNA oxidative damage [31,34]. Owing to its excellent formula compatibility, squalene is extensively incorporated into barrier repair creams and soothing lotions for sensitive and problematic skin, achieving integrated efficacy, including moisturization, anti-inflammation, and barrier reconstruction.

Beyond squalene, other Camellia triterpenoids and phenolic terpenoids exhibit potent antioxidant activity through direct scavenging of cutaneous ROS and inhibition of lipid peroxidation chain reactions, thereby protecting phospholipid bilayer cell membranes and collagen–elastin fiber structures from oxidative damage [35]. These triterpenoid components activate endogenous antioxidant enzyme systems such as superoxide dismutase (SOD) and glutathione peroxidase (GSH-Px), elevate the basal antioxidant capacity of the skin, and attenuate photo-aging and chronological aging manifestations, such as wrinkles, skin laxity, and pigmentary disorders [36]. In terms of anti-inflammatory mechanisms, triterpenoids negatively regulate the nuclear factor κB (NF-κB) and activator protein 1 (AP-1) signaling pathways, significantly downregulating the expression of pro-inflammatory cytokines TNF-α, IL-6, and IL-8 to inhibit cutaneous inflammatory cascades [35,37]. Moreover, triterpenic acids modulate sebaceous gland function, inhibit excessive sebum secretion, reduce pore size, decrease surface lipid accumulation, and prevent pore blockage, thus improving oily skin and acne-prone skin conditions [21].

Collectively, Camellia terpenoids including monoterpenes, sesquiterpenes, and triterpenoids achieve broad-spectrum skincare efficacy through multi-pathway synergistic regulation. All terpenoid subtypes exert antioxidant, anti-inflammatory, and pigmentation-regulating effects, while volatile terpenoids soothe sensitive skin, and triterpenoids, represented by squalene, are specialized in barrier repair and metabolic regulation [22]. In current cosmeceutical development, Camellia terpenoids are typically formulated in combination with polyphenol extracts, micronutrient derivatives, and phospholipid repair components to construct multifunctional formula systems, realizing comprehensive effects including free radical scavenging, anti-aging, antimicrobial activity, sebum regulation, and skin barrier protection, and they have broad application prospects in sensitive skin repair, inflammatory skin improvement, and anti-photo-aging skincare [22].

### 3.2. Tea Saponin

Tea saponins (Camellia triterpenoid saponins) are widely distributed in Camellia roots, stems, leaves, flowers, fruits and seeds, with varying content and composition among different plant parts. As natural products possessing diverse pharmacological activities, they demonstrate antibacterial, anti-inflammatory, antioxidant, and anticancer effects and inhibit alcohol absorption and cell proliferation [38]. Tea saponins comprise sapogenin, sugar moiety, and organic acid.

In skin cleansing applications, tea saponin functions as a natural non-ionic surfactant (pH 5–7, compatible with human skin) with minimal irritation. It gently eliminates dirt and excess sebum while preserving skin moisture, keeping skin hydrated post-cleansing and rendering it suitable for all skin types [39]. It also modulates sebaceous gland function, reducing excessive sebum production and adsorbing surface oil to prevent pore blockage, while its antimicrobial properties minimize odor and inflammatory stimuli resulting from lipid degradation to improve oily skin appearance [40].

Concerning anti-senescence and antioxidant effects, tea saponins demonstrate notable capability as efficacious hydroxyl radical scavengers within biological systems. Concurrently, they significantly enhance the catalytic activity of key antioxidant enzymes, particularly GSH-Px and SOD, whose activities are substantially increased under tea saponin influence [41]. Empirical evidence indicates that through the interplay between such enzymatic systems facilitated by tea saponin administration, oxidative injury is suppressed with enhanced efficiency, thereby exerting a retardatory effect on physiological processes underlying organismal aging [42].

Regarding anti-inflammatory potential, tea saponins exhibit significant activity characterized primarily by suppression of various pro-inflammatory mediators, which are critical contributors within inflammatory pathways [43]. Additionally, they possess broad-spectrum antimicrobial efficacy applicable to both bacterial and fungal species, with research demonstrating minimum inhibitory concentrations of 1 mg/mL for strains such as *Escherichia coli*, whereas 0.5 mg/mL effectiveness is observed against *Staphylococcus aureus*—the latter exhibiting parallel susceptibility when minimum bactericidal concentration values are established at 4 mg/mL. These bioactive compounds also demonstrate capacities for mitigation in murine experimental models wherein cutaneous lesions are induced by 2,4-dinitrochlorobenzene exposure: improvements occur in both epidermal restoration processes and, subsequently, in the resolution of compromised skin barriers observable post-challenge [44]. Furthermore, they promote keratinocyte proliferation and repair, increasing stratum corneum thickness and reducing inflammation-mediated barrier damage. When combined with other lipids, they synergistically maintain barrier integrity, alleviating redness and sensitivity [45].

In skin-whitening applications, tea saponin represents a valuable natural cosmetic ingredient through inhibiting tyrosinase, blocking 3,4-dihydroxyphenylalanine (DOPA)-to-dopaquinone conversion and suppressing melanin synthesis [46]. Characterized by its botanical origin, tea saponin exhibits biodegradability and remains free from pollutant-generating activities, attributes that align with both consumer preferences oriented toward green consumption and prevailing trajectories within cosmetic sector sustainability [9]. Within clinical dermatology, the pathognomonic signs of acne are dysfunctions centering upon overactive sebaceous gland secretion, the consequential occlusion observed at follicular levels, and an inflammatory milieu whose emergence is mediated through proliferative activity of *C. acnes*, and these pathological changes produce clinical lesions including comedones, papules and pustules in patients [47]. Tea saponin, in application, exhibits properties conducive to mild cleansing, characterized by effective lipid removal and enhanced clearance of follicular debris, with inhibition of *C. acnes*’ expansion alongside restriction of pro-inflammatory mediator release collectively resulting in attenuation of erythematous swelling localized at lesion sites; consistently, the cutaneous barrier integrity remains uncompromised in this context [39].

In contemporary dermatocosmetic formulations, tea saponin finds notable application across diverse product modalities, as evidenced in its incorporation within mild cleansing agents, oil-regulating anti-acne emulsions or solutions, and topicals designed for reparative intervention with inflammatory pathologies, as well as therapeutics targeting seborrheic dermatitis symptoms. When combined with actives from classes such as humectants, anti-inflammatory compounds, and compounds facilitating lipid barrier restitution, tea saponin imparts characteristics aligned with gentle detergent action, sebum regulation, antimicrobial potential, anti-inflammatory effects, and dermal barrier restoration [9].

Tea saponins represent mild, naturally derived surfactants from Camellia that preserve skin barrier lipids. Compared with other bioactive compounds, they exhibit unique penetration-enhancing properties that facilitate the transdermal delivery of other bioactive compounds. Concurrently, they thicken the stratum corneum and exert direct bactericidal effects by lysing bacterial cell membranes, thereby offering a barrier repair mechanism that is distinct from conventional lipid-based approaches. In terms of skin brightening, they inhibit DOPA conversion via a pathway that is mechanistically distinct from that of polyphenols [9].

### 3.3. Phenolic Compounds

#### 3.3.1. Phenolic Acids

Phenolic acids are low-molecular-weight organic phenolic acids widely distributed in Camellia flower extracts, with gallic acid serving as the most representative active monomer. Distinct from flavonoids and polymeric tannins in terms of molecular skeleton and chemical classification, gallic acid possesses abundant phenolic hydroxyl groups, enabling prominent free-radical-scavenging capacity to eliminate intracellular reactive oxygen species and alleviate cellular oxidative stress [48]. As a low-toxicity cosmetic-compatible phenolic component, gallic acid exerts core skin-brightening efficacy through inhibition of tyrosinase activity, thereby suppressing melanin biosynthesis and ameliorating UV-induced pigmentary abnormalities. In addition, it delivers auxiliary anti-inflammatory, antimicrobial and antioxidant effects, ameliorating dull skin texture and providing basic skin protection for daily skincare applications. Further its polyol structure also inhibits hyaluronidase activity, effectively preventing hyaluronic acid degradation and maintaining long-term epidermal hydration [49].

#### 3.3.2. Flavonoids

Flavonoids are a major class of plant secondary metabolites characterized by a conserved 2-phenylchromone skeleton, encompassing flavan-3-ols (catechins) and flavonols (rutin, quercetin, kaempferol), which collectively represent the phenolic bioactive compounds of Camellia flowers and leaves [50]. These compounds are categorized into monomeric flavonoids and flavonoid glycosides with distinct skincare mechanisms. Monomeric flavonoids represented by catechins exhibit excellent dual antioxidant capacity: they directly neutralize UV-triggered ROS to mitigate photodamage and suppress lipid peroxidation to stabilize cell membrane structure [51]. They also significantly inhibit the activity of skin aging-related enzymes including matrix metalloproteinase-1 (MMP-1) and elastase, reduce the enzymatic degradation of collagen and elastin, and delay cutaneous laxity and wrinkle formation [52]. Notably, catechins induce mild ROS production to activate cellular adaptive stress responses, enhancing skin tolerance against external oxidative insults [53]. With prominent anti-inflammatory and tyrosinase-inhibiting properties, monomeric flavonoids alleviate cutaneous erythema and pruritus via negative regulation of NF-κB and AP-1 signaling cascades and attenuate melanin synthesis to improve skin dullness and hyperpigmentation [54,55].

Flavonoid glycosides exemplified by rutin possess superior vascular regulatory and anti-sensitivity effects. They enhance capillary perfusion, reduce vascular permeability, and alleviate inflammatory swelling and burning sensations in sensitive skin [56]. In addition, flavonoids including quercetin and luteolin activate the nuclear factor erythroid 2-related factor 2 (Nrf2)/heme oxygenase-1 (HO-1) antioxidant axis, upregulate endogenous antioxidant enzymes such as SOD and GSH-Px, and inhibit nicotinamide adenine dinucleotide phosphate (NADPH) oxidase activity, comprehensively strengthening skin antioxidant defense [57]. They also block NF-κB, MAPK and Janus kinase/signal transducer and activator of transcription (JAK/STAT) immune signaling pathways, suppress the expression of pro-inflammatory mediators including cyclooxygenase 2 (COX-2) and inducible nitric oxide synthase (iNOS), and alleviate acute and chronic cutaneous inflammation [57,58,59]. Functionally, flavonoids promote keratinocyte proliferation and differentiation, upregulate ceramide and cholesterol synthesis to repair impaired skin barriers, and modulate aquaporin expression to maintain epidermal hydration [60]. They exert targeted improvement on melasma, post-inflammatory hyperpigmentation (PIH), rosacea and acne lesions, and are widely formulated into anti-aging, brightening and soothing skincare products [54,55]. Additionally, flavonoids bind to skin proteins through hydrophobic interaction and hydrogen bonding to refine pores, alleviate oily skin sebum over-secretion, and maintain long-term skin moisturization [49,61].

#### 3.3.3. Tannins

Tannins are high-molecular-weight polymeric polyphenols mainly consisting of hydrolysable and condensed tannin oligomers, which are uniquely differentiated from low-molecular-weight phenolic acids and flavonoids in molecular structure and skincare characteristics. As abundant bioactive compounds in Camellia species, tannins contain extensive hydroxyl functional groups and exert potent antioxidant effects through electron donation to scavenge ROS and reactive nitrogen species (RNS), terminating continuous oxidative chain reactions in skin tissues [62]. Distinctive from other phenolic substances, tannins possess unique protein precipitation and astringent properties: they form stable hydrogen-bonded complexes with epidermal surface proteins, enabling instantaneous skin tightening, pore refinement and fine wrinkle reduction, which effectively ameliorates sagging and rough skin texture [63].

Tannins exert anti-inflammatory effects primarily through inhibition of NF-κB and toll-like receptor 4 (TLR4) signaling pathways, downregulating the expression of pro-inflammatory cytokines IL-1, IL-6, TNF-α and upregulating the anti-inflammatory cytokine IL-10, thereby fundamentally alleviating cutaneous inflammatory responses [64,65]. They also exhibit broad-spectrum antimicrobial activity against common skin bacteria and fungi at cosmetic-grade concentrations without inducing cytotoxicity to normal human skin cells, rendering them suitable for anti-irritation and anti-aging skincare development [66,67]. Another distinctive advantage of tannins lies in their prominent ultraviolet shielding performance, which can absorb over 98% of incident UV radiation, effectively reducing solar erythema risk and cumulative photo-aging damage, realizing synergistic photoprotection for skin [63]. In terms of brightening and moisturizing efficacy, tannins inhibit tyrosinase activity to suppress de novo melanin synthesis, accelerate the turnover of pigmented keratinocytes to fade existing hyperpigmentation [68], and inhibit hyaluronidase activity to preserve dermal hyaluronic acid, forming a hydrophilic protective film on the skin surface to achieve long-lasting deep moisturization [63].

### 3.4. Sterols

Phytosterols are predominantly distributed in Camellia seeds, with minor quantities found in flowers and leaves. These compounds feature antioxidant, anti-inflammatory, antibacterial and skin-brightening effects, effectively repairing damaged skin barriers and delaying skin aging, thereby serving as ideal raw materials for sensitive skincare and anti-aging products.

Sterols exhibit molecular resemblance to endogenous cholesterol, a property that facilitates their progressive assimilation into cutaneous cellular frameworks, particularly within regions characterized by a paucity of physiological constituents [69]. When concentrations within the plasma membrane exceed 10%, synergistic interactions occur with sphingolipids, facilitating the assembly of specialized lipid microdomains known as rafts. These domains are central to membrane stabilization and dynamic viscosity; they are essential for protein functionality pertaining to the epidermal barrier, thereby mediating homeostatic restoration [70]. Owing to the marked lipophilic attributes of sterols, they form an occlusive film on the stratum corneum surface, functioning to impede transepidermal water loss, thus achieving attenuation of such losses [71]. Within this context, sterol esters further support repair of intercellular matrix lipids, intrinsically maintain hydration, and exhibit softening effects on keratinization [72].

Sterol compounds extend beyond mere structural reinforcement, demonstrating capabilities against microbial proliferation, encompassing both bacterial and fungal species. Additionally, anti-inflammatory responses, antipyretic modulation, and ulcer attenuation are among the pharmacodynamic characteristics observed in experimental models [73]. In individuals manifesting heightened dermal sensitivity or inflammatory reactivity, symptomatic irritation is mitigated through such actions. The underlying mechanisms involve suppression of NF-κB initiation cascades; consequentially, diminished secretion rates for principal pro-inflammatory effectors, including tumor necrosis factor alpha and interleukin family mediators, are observed [74]. Furthermore, regulation is exerted over immune cell behavior within cutaneous tissue environments, whereby chronic pathological states characterized by persistent erythema or pruritic manifestations experience measurable relief [75].

The antioxidant properties attributed to sterols operate through a dual mechanism: direct reactivity with ROS, alongside enhancement of endogenous enzymatic pathways, including those governed by SOD and GSH-Px, thereby facilitating attenuation of oxidative damage [76]. Consequently, suppression characterizes the progression of inflammation-associated lipid peroxidation—a relationship evident upon examination of relevant biochemical cascades that are interrupted within such contexts. Notably, stabilization afforded by sterols to membrane lipid matrices, particularly in vascular endothelial cells and hepatocytic populations, proves instrumental for defensive mechanisms deployed against ROS-provoked structural injury [77]. Thus, preservation of oil integrity from rancidification becomes apparent, alongside broader maintenance of systemic redox homeostasis, which, when extrapolated across organismal physiology, suggests a possible reduction in incidences of diseases linked to heightened oxidative stress states [78]. Regarding integumentary system manifestations, this multifaceted antioxidative action manifests as increased resistance to exogenous environmental insults, a phenomenon that aligns concomitantly with observable deceleration in cutaneous senescence.

Based on accumulated empirical studies, sterolic compounds exhibit significant inhibitory effects on human leukocyte elastase (HLE) [79]. Elastase plays a critical role in skin turnover under physiological conditions, facilitating dermal metabolic turnover through the clearance of denatured ECM proteins. Marked deviations from this regulated activity, an aberrance noted in pathophysiological states, affords a scenario within which persistent inflammation and progressive proteolysis of structural constituents such as elastin and collagen ensue; thus, integral support structures for cutaneous pliancy and resistance are compromised [80,81]. This implies that intervention with sterol-based inhibitors maintains tensile skin properties, retards processes underlying wrinkle ontogeny through preservation of stromal flexibility, and provides mitigating capacity against tissue injury associated with inflammatory phenomena [82,83]. Within clinical practice, sterols emerge as agents targeting the fundamental dysregulations intrinsic to prevalent inflammatory dermatoses. As manifested in AD, wherein inherent deficits in epidermal lipid components and persistent inflammatory sequelae prevail, compensatory restoration of barrier lipids by sterols is observable, with attenuation of mediator-driven inflammation thereby facilitating pruritus alleviation and episodic exacerbation minimization. This pattern, evident across multiple documented outcomes, illustrates the dualistic modulation—replenishing the structural deficit while simultaneously interfering with pathogenic signaling routes—which underpins the therapeutic efficacies ascribed to sterol-based interventions among afflicted individuals [74]. Discernible from the prevailing utilization landscape of sterols is their notable inclusion within diverse dermatological formulations, including creams for barrier restitution, lotions with anti-inflammatory functions, moisturizing unguents, age-mitigative assemblages, and preparations tailored to dermally sensitive cohorts.

Sterols share a high structural similarity with human cholesterol, enabling them to embed into and repair lipid rafts on cell membranes—a property not commonly observed in oils or polyphenols. Specifically, they inhibit human leukocyte elastase, thereby reducing elastin degradation and targeting sagging and fine lines. Additionally, phytosterols bidirectionally regulate skin immunity, alleviating chronic recurrent sensitive dermatitis. This immunomodulatory repair mechanism is distinct from that of other Camellia-derived actives, which primarily provide anti-inflammatory or lipid-replenishing benefits.

### 3.5. Amino Acids and Proteins

Camellia seed meal, a byproduct generated during Camellia seed oil extraction, serves as an abundant source of proteins and bioactive peptides. These peptide substances possess outstanding biochemical activity and have attracted extensive research interest.

Numerous analytical studies indicate that the antioxidative potencies of these peptide molecular fractions sometimes surpass the comparable activities of saponin compounds present in the same matrix. Cell-free assay paradigms demonstrate that oxidative perturbations manifesting as elevated intracellular ROS and malondialdehyde (MDA) concentrations can be mitigated following exposure to proteinaceous extracts derived from Camellia seed meal; concomitant elevation in SOD, as well as GSH-Px enzymatic functions, substantiates the preservation effects on redox equilibria inherent to biological systems [84]. Noteworthy are the mechanistic insights into the operational scheme of this antioxidant effect: herein, particular amino acid moieties—tyrosine, histidine, and cysteine identified among them—fulfill the electron or hydrogen donor roles requisite for direct radical quenching interactions. Activation potential toward the Nrf2/HO-1 transduction axis evidenced in select studies indicates that upregulation of endogenous antioxidase constituents, accountable post hoc for cytostructural safeguarding, occurs through protein-mediated pathway initiation [44].

Of considerable relevance within the realm of cutaneous science, amino acids emerge as multifaceted agents conferring a spectrum of physiological advantages. Constituting key components integral to the natural moisturizing factor (NMF), it is through their molecular presence in the upper layers of the epidermis that homeostasis of hydration in the stratum corneum is sustained, with attenuation evident in transepidermal water efflux; from this follows the observable maintenance of integumentary softness [85]. Salient among these compounds appear serine and arginine, whose pronounced hygroscopicity imparts further reinforcement to barrier functionality. Demonstrated by investigations into reparative processes, wound closure undergoes facilitation via arginine and glutamine, phenomena simultaneously accompanied by modulation on inflammatory pathways and the inducement—traceable at a biomolecular level—of dermal matrix protein synthesis, notably involving collagen and elastin, upon which modifications in biomechanical resilience are dependent [86].

For AD, amino acids directly supply critical precursors for barrier synthesis, supporting stratum corneum repair and lipid production. The suppression, by anti-inflammatory mechanisms, of the liberation undergone by inflammatory mediators manifests itself in an attenuation observable within pruritic sensations and a diminution evident in exacerbations precipitated through extrinsically acting triggers [87]. Asteatotic eczema can be traced to the insubstantial secretory performance inherent in sebaceous glands accompanied by nutritive insufficiencies (a paucity discernible notably in amino acids), leading ultimately to impairment characteristic of the cutaneous barrier so that dryness becomes pronounced, textural irregularities ensue, and desquamative phenomena persist. Recognizable among hydrophilic functionalities possessed by amino acid moieties is their interactive capacity with stratum corneum water content: with this potential for moisture restitution and augmentational retention being invested directly upon the uppermost epidermal layers [85], reversals in barrier deterioration acquire support. Materials provided thereby are essential for reconstructing structural attributes of the stratum corneum, whose reparative processes, contingent on such substrates, see both scaling and subjective itch modulation. In clinical instances typifying contact dermatitis (CD), facilitation emerges for swift reinstatement regarding the disrupted skin defense apparatus, as evidenced by decreased persistence characterizing deleterious irritants at lesion sites. Alleviation detectable in inflammation severity, pacification noticeable in noxious cutaneous sensation, stability measurable in pH levels approaching normalization, and enhancement visible in wound convalescence may all be corroborated by outcomes encountered contextually [88,89].

In light of these functionalities, frequent is the incorporation of amino acids into formulations for gentle cleansers, hydrating lotions, creams directed at barrier repair, and skincare products tailored towards sensitive skin. Combined with substances including ceramides and panthenol, together with phytosterols, such formulations are deployed to patient populations exhibiting conditions typified by AD, asteatotic eczema or even CD, which encompass well-generalized phenomena of dryness and cutaneous hypersensitivity [90]. It might be observed from such strategized combinations that cleansing characterized by mildness, hydration maintained over time, restoration processes targeting disrupted epidermal barriers, and attenuation responses of inflammation proceed to manifest in tandem. Thus, a dynamic equilibrium is achieved wherein perceived reparative effectualness meets therapeutic gentleness, illustrative of a nuanced approach whereby efficacy does not preclude tolerability [90].

The uniqueness of amino acids is manifested in the core constituents of the stratum corneum’s NMF, providing endogenous hydration that contrasts with the occlusive mechanism of oils. Among them, arginine directly stimulates collagen synthesis and accelerates wound healing. Unlike lipids, amino acids pose no pore-clogging risk and are well tolerated by highly sensitive skin. Furthermore, they supply essential precursors for barrier replenishment, operating via a repair pathway distinct from that of lipid-based actives [91].

### 3.6. Fatty Acids (FAs)

FAs are primarily accumulated in Camellia seeds, and Camellia seed oil is rich in unsaturated fatty acids (UFAs). Oleic acid and linoleic acid account for over 90% of total FAs in seed oil, whereas only trace amounts of FAs exist in other plant tissues, making seeds the dominant raw material for FA extraction [92,93]. Functionally, FAs possess prominent lipophilic properties and strong skin affinity, acting as core components that construct the skin lipid barrier.

FAs serve as integral components within the lipidic matrix of the stratum corneum, functioning synergistically with cholesterol and ceramides—elements whose cooperative engagement is consistently emphasized in contemporary dermatological research [94]. Upon topical administration, exogenous FAs supplement essential lipids deficient in compromised epidermal zones; consequently, intercellular spaces previously rendered vulnerable through depletion are reconstituted, restoring barrier integrity. This process confers enhanced protection against external irritants, while diminished transepidermal water loss provides measurable evidence of amelioration particularly beneficial for regions with impaired barrier function. The inherent lipophilicity of FA molecular structures facilitates their pronounced propensity for coalescence with endogenous skin lipid matrices, resulting in the formation of a permeable occlusive film capable of retarding surface evaporation rates, as demonstrated in clinical studies [95].

Integration of UFAs and their derivatives within physiological contexts revealed that pronounced anti-inflammatory capacities manifest during wound healing processes. Distinct UFA subtypes mediate modification of inflammatory signaling axes, each exerting specialized molecular influences. Specifically, omega-3 polyunsaturated FAs, including α-linolenic acid (ALA), eicosapentaenoic acid (EPA), and docosahexaenoic acid (DHA), exert competitive inhibition that occurs at the cyclooxygenase (COX) and lipoxygenase (LOX) loci, thereby suppressing pro-inflammatory eicosanoid synthesis while enhancing the abundance of mediators classified as anti-inflammatory [96]. Metabolic conversion of DHA yields resolvins, which regulate inflammation through restriction of neutrophil extravasation and attenuation of cytokine production [97]. Additionally, EPA and DHA directly target peroxisome proliferator-activated receptor gamma (PPAR-γ), inducing dampening effects on transcriptional drivers such as NF-κB [98] and illustrating the complex network characterizing UFA-mediated immunomodulation. Beyond biochemical signaling, UFA bioactivity encompasses preservation of membrane biophysics and alleviation of oxidative stress-induced inflammatory sequelae [99]. Accompanying this, barrier reconstitution contributes to restriction of noxious agent ingress into cutaneous tissues. Consequently, manifestations including erythema, burning discomfort, and pruritus associated with inflammatory dermatological conditions can be efficiently moderated through therapeutic exploitation of these UFA properties.

Within the clinical framework of AD, FAs exert a dual mechanistic influence: directly supplying lipidic barrier restitution to cutaneous strata while simultaneously inhibiting inflammatory mediator release—an attenuation that yields reduced pruritus and decreased exacerbation frequency. Clinical evidence positions FAs as pivotal modulatory agents in epidermal restoration for AD patients [99]. When combined with ceramides and cholesterol, restorative outcomes are enhanced. In asteatotic eczema, resulting from insufficient sebaceous gland secretion and lipid depletion, FAs form a surface-occlusive film to retain moisture, soften the stratum corneum to reduce scaling, and supply barrier precursors for repair, thereby alleviating dryness, tightness, and pruritus [100]. Regarding CD, FAs rapidly restore integrity to the disrupted stratum corneum barrier—a layer frequently impaired in CD [94]. This restoration process mitigates lingering irritant substances on the cutaneous surface, resulting in substantial diminishment of irritative sequelae. Clinical applications demonstrate that FA-induced anti-inflammatory effects coincide with accelerated reparative responses at lesional sites, leading to notable abatement of sensitivity across treated epidermal zones.

FA-containing formulations have been extensively incorporated into barrier-restorative emulsions, hydrating topical agents, and dermal solutions designed for sensitive skin. These formulations frequently integrate constituents such as ceramides, cholesterol derivatives, and panthenol—combinations specifically targeted toward dermatological manifestations including AD, asteatotic eczema, CD, along with physiologically dry, structurally compromised, or aging skin presentations [91]. Current research reveals multifaceted functionalities inherent to these blends: beyond supporting epidermal homeostasis restoration and enhancing transepidermal moisture retention, they also provide anti-inflammatory mitigation and cutaneous texture softening. Thus, a notable equilibrium emerges between gentle tolerability and cumulative reparative efficacy during extended applications [90].

The distinction between FAs and other bioactive compounds lies in FAs being essential structural components of the stratum corneum lipid barrier. Notably, omega fatty acids resolve chronic inflammation through the generation of specialized pro-resolving mediators (e.g., resolvins), a mechanism distinct from conventional actives that merely suppress inflammatory cytokines [101]. Additionally, FAs contribute to sebum composition balance, rendering them particularly beneficial for combination skin characterized by superficial oiliness coexisting with underlying dryness, offering a regulatory effect that extends beyond temporary oil control. Ultimately, along with cholesterol and ceramides, FAs are indispensable for fully reconstituting the intact skin lipid barrier [102].

## 4. Summary and Perspectives

Camellia species represent botanical entities distinguished by dual ecological and economic capacities, abundant in multiple classes of bioactive compounds including flavonoids, saponins, polyphenols, and terpenoids (Table 1). Extant research indicates that these molecular groups encompass compounds characterized by antioxidant, anti-inflammatory, antimicrobial, moisturizing, and skin chromatic modulation properties. Consequently, these characteristics have driven their widespread adoption as foundational intermediates throughout research and development endeavors spanning cosmetics, pharmaceuticals, and diverse nutraceutical sectors [44]. Considerable international advancements have been made regarding fractionation, characterization, activity validation, and preliminary translational applications of Camellia-derived agents. This body of work confirms their distinct advantages in dermatological care and significant potential across multiple application domains; however, the complete development pipeline—from basic research to industrial commercialization—continues to encounter numerous bottlenecks [103].

Several key constraints currently impede the advancement of Camellia bioactive research. Significant heterogeneity exists in both the content and spectrum of bioactive compounds among Camellia varieties, with notable distinctions attributable to disparities in environmental growth parameters and differentiation within plant anatomical structures. Within this variability, regulatory mechanisms governing such biochemical diversifications remain only partially elucidated; consequently, these deficiencies in mechanistic understanding limit the methodical optimization of agronomic management and precise stratification when sourcing superior-grade raw materials for downstream development. These knowledge gaps restrict advancements in directed breeding strategies and targeted improvement efforts within Camellia genetic resources [104].

Extraction methodologies continue to rely predominantly on conventional solvent-based techniques such as ethanol reflux, which often exhibit inefficiency, leave solvent residues, and risk degrading heat-sensitive compounds including gallic acid and squalene. Although greener alternatives such as supercritical fluid and microwave-assisted extraction exist, their industrial-scale optimization remains preliminary due to high equipment costs and unstable batch yields [105]. In terms of application and translation, current uses of Camellia bioactives are largely confined to single compounds or crude extracts. Research into the synergistic interplay between different components remains limited, slowing the development of sophisticated formulations and preventing the full exploitation of Camellia’s “multi component, multi target” potential [5]. Studies involving numerous bioactives reside primarily at preclinical strata, with investigations restricted to cellular frameworks or animal models. Evidence delineating metabolic trajectories in humans, longitudinal safety margins, and tangible clinical benefits is shown, thereby, to be markedly circumscribed; representative bioactive compounds including Camellia tannins and triterpenoid saponins lack complete human transdermal metabolism and long-term skin safety evaluation data. These deficiencies impede utilization within pharmaceutical formulations intended for human use and high-value cosmetic industry applications [44].

**Table 1:** Camellia Bioactive Compounds: Their Bioactivities, Sources and Applications.

Categories of Bioactive Compounds	Main Biological Activity	Source Part	Types of Skin Diseases	References
Terpene	Antioxidant and intracellular ROS scavenging Anti-inflammatory and skin inflammation alleviation Soothing, conditioning, and skin barrier repair	Leaves Fruits Bark Seeds	Infectious skin diseases Inflammatory skin diseases Skin photo-aging Sensitive skin Pigmentary skin disorders	[19,21,22,29,35,37,79,106,107]
Saponins	Gentle cleansing and surface tension reduction Skin absorption promotion and efficacy enhancement of other bioactive compounds Antibacterial effects via disruption of pathogenic bacterial membrane integrity Skin stratum corneum moisturization and water content improvement	Leaves Flowers Seeds	Acne Seborrheic dermatitis Contact dermatitis Tinea manus et pedis Dry skin disorders	[9,19,38,42,43,44,45,46,47,107,108]
Phenolic Acids	Antioxidation by scavenging ROS Inhibit tyrosinase for skin brightening Anti-inflammatory and antibacterial effects Suppress hyaluronidase to retain moisture	Leaves Flowers	Skin photo-aging Pigmentary skin disorders Inflammatory skin diseases Sensitive skin disorders Seborrheic dermatitis	[48,49]
Flavonoid	Antioxidant activity and enhancement of endogenous antioxidant enzymes Anti-inflammatory effects via regulation of the NF-κB inflammatory signaling pathway Skin-whitening properties through competitive inhibition of tyrosinase Soothe skin sensitivity and alleviation of ultraviolet-induced skin damage	Leaves Flowers Bark	Skin photo-aging Pigmentary skin disorders Inflammatory skin diseases Sensitive skin Seborrheic dermatitis	[50,51,52,53,54,55,56,58,59,109,110,111]
Tannin	Skin free radical scavenging, lipid peroxidation inhibition, and aging delay Skin protein binding to form a protective film, pore shrinkage, excessive sebum secretion reduction, inflammatory factor release inhibition, and skin inflammatory response alleviation Antibacterial and bacteriostatic effects for reducing the risk of skin infections Tyrosinase activity inhibition, melanin synthesis and transport blockade, and PIH reduction for skin brightening	Leaves Flowers Fruits Bark	Acne Seborrheic dermatitis Pigmentary skin disorders Contact dermatitis Skin photo-aging	[62,66,67,68,112]
Sterol	Lipid component supplementation, lipid bilayer repair, moisture-locking capacity enhancement, and resistance improvement to external stimuli Inflammatory factor release inhibition and skin inflammatory response regulation Lipid peroxidation free radical scavenging, skin cell damage reduction, and skin aging delay Skin lipid metabolism balance and skin condition improvement	Leaves Flowers Seeds	Barrier-impaired skin disorders Sensitive skin Seborrheic dermatitis Skin photo-aging Winter pruritus	[45,69,71,72,73,74,75,76,77,78,79,113,114,115]
Amino acids and proteins	Moisture replenishment and locking for dry and rough skin improvement Skin stratum corneum lipid synthesis participation and damaged barrier repair Skin inflammatory signaling pathway regulation and inflammatory factor release inhibition Skin elasticity enhancement and aging manifestation delay (e.g., wrinkles, sagging)	Leaves Flowers Seeds	Barrier-impaired skin disorders Sensitive skin Dry skin disorders Skin photo-aging Acne	[61,82,84,85,86,87,88,89,116,117]
Fatty acids	Skin barrier repair and stratum corneum lipid supplementation Moisturization and water locking for reduction in skin moisture loss Anti-inflammatory effects and skin lipid metabolism regulation Skin nourishment and roughness improvement	Seeds (Camellia seed oil)	Barrier-impaired skin Dry skin Inflammatory skin Skin photo-aging Acne	[81,94,96,99,118,119]

On the prospective trajectory concerning Camellia-derived bioactive compounds, three principal axes—precision-targeting optimization, environmentally adaptive processual methodologies, and interfacing translational industrialization—emerge as dominant. Into molecular elucidation efforts, an imperative is discernible for integrated deployment of contemporary omics stratagems in conjunction with diversified molecular biology modalities, whereby complex mechanistic pathways implicated by these compounds may be mapped out at both cellular and organismal levels. For procedural enhancements in extraction regimes, significant emphasis should be placed upon refinement of efficiency parameters alongside minimization of ecological repercussions attendant upon processing routines associated with Camellia matrices. Examination of sectorial convergence reveals that interdisciplinary reinforcement constitutes a catalyst for proliferating application arenas into functional alimentary products, topical cosmetological innovations, and precisely formulated pharmaceutical preparations. Through expanding investigatory sophistication and deepening institutional alignment between academic and commercial spheres, resource potential inhering to the genus Camellia stands positioned for transformative influence in both biomedical health promotion and sustainable biosphere stewardship, with observable momentum fostered by progressive industrial synergy.

## Data Availability

No new data were created or analyzed in this study. Data sharing is not applicable to this article.

## Abbreviations

The following abbreviations are used in this manuscript:

**Abbreviation**	**Full Name**
AD	Atopic Dermatitis
ALA	α-linolenic Acid
AP-1	Activator Protein 1
*C. acnes*	*Cutibacterium acnes*
cAMP	Cyclic Adenosine Monophosphate
CD	Contact Dermatitis
COX	Cyclooxygenase
COX-2	Cyclooxygenase 2
DHA	Docosahexaenoic Acid
DOPA	3,4-Dihydroxyphenylalanine
ECM	Extracellular Matrix
EPA	Eicosapentaenoic Acid
FA	Fatty Acid
GSH-Px	Glutathione Peroxidase
HLE	Human Leukocyte Elastase
HO-1	Heme Oxygenase-1
IL-1	Interleukin-1
IL-10	Interleukin-10
IL-6	Interleukin-6
IL-8	Interleukin-8
iNOS	Inducible Nitric Oxide Synthase
JAK/STAT	Janus Kinase/Signal Transducer and Activator of Transcription
LOX	Lipoxygenase
MAPK	Mitogen-Activated Protein Kinase
MDA	Malondialdehyde
MITF	Microphthalmia-Associated Transcription Factor
MMP-1	Matrix Metalloproteinase 1
NADPH	Nicotinamide Adenine Dinucleotide Phosphate
NF-κB	Nuclear Factor κB
NMF	Natural Moisturizing Factor
Nrf2	Nuclear Factor Erythroid 2-Related Factor 2
PIH	Post-inflammatory Hyperpigmentation
PPAR-γ	Peroxisome Proliferator-Activated Receptor γ
RNS	Reactive Nitrogen Species
ROS	Reactive Oxygen Species
SOD	Superoxide Dismutase
TLR4	Toll-Like Receptor 4
TNF-α	Tumor Necrosis Factor-α
UFA	Unsaturated Fatty Acid
